# Analysis of Multidrug Transporter in Living Cells. Inhibition of P-glycoprotein-mediated Efflux of Anthracyclines by Ionophores

**DOI:** 10.1155/MBD.1994.175

**Published:** 1994

**Authors:** Marie-Nicole Borrel, Elene Pereira, Marina Fiallo, Arlette Garnier-Suillerot

**Affiliations:** 1 Laboratoire de Chimie Bioinorganique LPCB (URA CNRS 198) Université de Paris Nord 74 rue Marcel Cachin Bobigny F- 93012 France

## Abstract

One of the major obstacles of chemotherapy is that, after repeated treatments, cellular resistance to the drug appears. The problem is that the tumor cells become resistant not only to the drugs which have been used during the treatment but also to other drugs which are structurally and functionally unrelated. This is termed ‘multidrug resistance’ (MDR). MDR is frequently associated with decreased drug accumulation resulting from enhanced drug efflux. This is correlated with the presence of a membrane protein, P-glycoprotein, which pumps a wide variety of drugs out of cells thus reducing their toxicity. The search for molecules able to reverse MDR is very important. We here report that mobile ionophores such as valinomycin, nonactin, nigericin, monensin, calcimycin, lasalocid inhibit the efflux of anthracycline by P-glycoprotein whereas, channel-forming ionophores such as gramicidin do not. Cyclosporin which is also a strong Ca^2+^ chelating agent also inhibits the P-glycoprotein-mediated efflux of anthracycline.


### ANALYSIS OF MULTIDRUG TRANSPORTER IN LIVING CELLS. INHIBITION OF P-GLYCOPROTEIN-MEDIATED EFFLUX OF ANTHRACYCLINES BY IONOPHORES

Marie-Nicole Borrel, Elene Pereira, Marina Fiallo and Arlette Garnier-Suillerot*

Laboratoire de Chimie Bioinorganique, LPCB (URA CNRS 198), Université de Paris Nord,
74 rue Marcel Cachin, F - 93012 Bobigny, France

*to whom correspondence should be addressed

**Abstract**

One of the major obstacles of chemotherapy is that, after repeated treatments, cellular resistance to the drug appears. The problem is that the tumor cells become resistant not only to the drugs which have been used during the treatment but also to other drugs which are structurally and functionally unrelated. This is termed 'multidrug resistance' (MDR). MDR is frequently associated with decreased drug accumulation resulting from enhanced drug efflux. This is correlated with the presence of a membrane protein, P-glycoprotein, which pumps a wide variety of drugs out of cells thus reducing their toxicity. The search for molecules able to reverse MDR is very important. We here report that mobile ionophores such as valinomycin, nonactin, nigericin, monensin, calcimycin, lasalocid inhibit the efflux of anthracycline by P-glycoprotein whereas, channel-forming ionophores such as gramicidin do not. Cyclosporin which is also a strong $Ca^{2+}$ chelating agent also inhibits the P-glycoprotein-mediated efflux of anthracycline.

One of the major obstacles of chemotherapy is that, after repeated treatments, cellular resistance to the drug appears. It has been estimated that among the one million deaths per year due to cancer in Europe, 90% of them were influenced by the problem of drug resistance to chemotherapeutic agents. The problem is that the tumor cells become resistant not only to the drugs which have been used during the treatment but also to other drugs which are structurally and functionally unrelated.

This is termed 'multidrug resistance' (MDR) [1,2].

MDR is a now well-characterised phenomenon. The mechanisms of MDR are far from been elucidated and, in fact, modifications of several cellular functions have been observed. Thus, MDR is frequently associated with decreased drug accumulation resulting from enhanced drug efflux. This is correlated with the presence of a membrane protein, P-glycoprotein, which pumps a wide variety of drugs out of cells thus reducing their toxicity [3-7]. This drug efflux is ATP-dependent.

Genetic analysis suggests that P-gp is a member of a family of membrane associated transport proteins which are involved in multidrug resistance, malarial chloroquine resistance by Plasmodium falciparum, cystic fibrosis, peptide transport, heavy metal resistance and bacterial transport processes [8}. P-gp is also present in several normal tissues [9]. However, to date no naturally occurring substrates have been identified for P-gp.

The mechanism of the transport process is not known for any of the transporters of the ABC super family, including one reconstituted in artificial vesicles. The hydrolysis of ATP apparently provides the energy for drug extrusion from the cell.

Perhaps the most puzzling question related to P-gp function is the unique ability of P-gp to recognise and transport a great number of apparently unrelated compounds. This transport can be inhibited by various compounds. It has been suggested that the minimum set of structural and functional features required for modulator binding to P-gp would be two planar aromatic domains and a nitrogen atom [10].

To analyse the function of the multidrug transporter in living cells, we have developed a method with which it is possible to follow the uptake of fluorescent drugs, such as anthracyclines, by cells, continuously, as incubation of the drug with the cells proceeds [11-13] This method does not perturb the drug distribution in the various cell compartments and is a simple and rapid technics for measuring intracellular drug accumulation, membrane transport rates or changes in membrane transport rates.

In the course of our studies concerning the effect of membrane potential on the uptake and release of anthracycline derivatives by drug sensitive and resistant cells we were surprised to observe that ionophores such as nigericin, valinomycin etc which are currently used to abolish the transmembrane potential were in fact able to inhibit the P-glycoprotein-mediated efflux of anthracycline. This lead us to systematically investigate the effect of ionophores on the P-gp-mediated efflux of 4'-o-tetrahydropyranyl-adriamycin (THP-adriamycin).in resistant K562 (an erythroleukemia cells line).

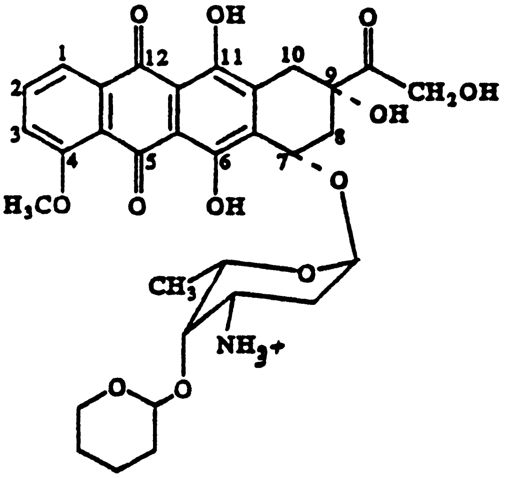

The possibility that some ionophores could modulate multidrug resistance has been reported. However, considerable confusion exists regarding the mechanism(s) by which ionophores bring about effects in resistant (and parent) cells: the modulation of adriamycin resistance in Chinese hamster ovary cells has been reported and the authors concluded that valinomycin exerts its actions by mechanism(s) other than increasing intracellular accumulation of drug [14]. Also, Crifo et al. have proposed that the adriamycin toxicity in a adriamycin-resistant human cancer cell line can be enhanced by very low concentration of valinomycin [15]. Recent studies using carboxylic ionophores such as monensin and nigericin have indicated that these agents can modulate anthracycline toxicity apparently by causing an enhancement of anthracycline accumulation [16,17]. It has also been noticed that the cytotoxic action of drugs as monensin [18] and nigericin ([19] may be assigned in the decrease cellular ATP levels.

In order to check what was the action of these ionophores on the P-gp-mediated efflux of drugs and thus on the intracellular drug accumulation, we have determined the experimental conditions in which the additions of these ionophores would lead neither to an intracellular pH modification nor to a plasma membrane potential variation. For this purpose we have used sensitive cells and checked the effects of ionophores on the intracellular accumulation of THP-adriamycin. In $Na^+$-containing hepes buffer the addition of nigericin and lasalocid yielded an increase of the drug accumulation due to a decrease of the intracellular pH ($pH_i$), whereas the addition of monensin yielded a decrease of the drug accumulation due to an increase of $pH_i$. However, in $K^+$-containing hepes buffer, these three ionophores are not able to modify $pH_i$ and by so far do not yield a modification of the THP-adriamycin accumulation. The other ionophores that we have tested, i.e. calcimycin, valinomycin, nonactin, as well as cyclosporin do not yield modification of the THP-adriamycin accumulation. either in $Na^+$ or $K^+$-containing hepes buffer. We have also checked that $pH_i$ was the same in both sensitive and resistant cells [12] . On the other hand the membrane potential is the same for type of cells and has no effect on the THP-adriamycin accumulation.

In the case of resistant cells we have observed that the addition of these ionophores lead to an increase of THP-adriamycin accumulation. For instance, when $10^6$cells/ml are incubated with 1μM THP-adriamycin, the overall concentration of drug bound to the nucleus at the steady state is $(C_n)_S$=0.54μM and $(C_n)_R$=0.25μM for sensitive and resistant cells respectively.The addition of ionophore to the resistant cells gives rise to an increase of $(C_n)_R$. If $(C_n)_{Ri}$ stands for the overall concentration of drug bound to the nucleus of resistant cells in the presence of the concentration [I] of ionophore we have the following equation.

$$(C_n)_{Ri} = (C_n)_R + [(C_n)_S - (C_n)_R]\alpha$$

where $\alpha$ is comprised between 0 (in the absence of ionophore) and 1.

Under our experimental conditions, the amount of ionophores required to obtain $\alpha$=0.5 is 0.3μM (nigericin), 0.4μM (lasalocid), 1.1μM (monensin), 1.2μM (calcimycin), 0.7μM (valinomycin), 0.3μM (nonactin). In the case of cyclosporin ,which is a now a well recognised MDR reverting agent,

0.3μM of product is required to obtained $\alpha$=o.5.For a sake of comparison we can add that, under our experimental conditions, 2μM verapamil, the well-known MDR reversing agent, are required to obtain $\alpha$=o.5. Under our experimental conditions, gramicidin has no effect on the intracellular accumulation of THP-adriamycin accumulation. On the other hand, we have checked that all these ionophores do not lead to a decrease of the intracellular ATP level.

Ionophores are a heterogeneous group of antibiotic which have the common propertiy of altering membrane permeability towards small inorganic ions. There are two basic types: (a) channel-forming ionophores (e.g.:gramicidin) that formed water-filled transmembrane channels or pores of narrow diameter, which permit the movement of ions and small molecules; (b) mobile ionophores that binds metal ions or organic cations to form lipid soluble complexes, which can then shuttle back and forth across the cell membrane. The second class can be divided in two groups: those, such as valinomycin and nonactin, which transport cations as lipid-soluble charged complexes and those, such as nigericin , monensin, lasalocid, calcimycin, which contain a charged carboxyl group and transports cations as lipid-soluble, electrically neutral complex [20].

Cyclosporin A is not considered as a mobile ionophore however it shared with this class of compounds a certain number of properties: its a strong metal chelating agent [21], it can affect the physico-chemical properties of the plasma membrane it has been shown to bind to phospholipid vesicles ([22] and depolarise cytoplasmic membrane potentials [23]. The first report of a preferential effect of CsA in resistant versus sensitive tumour cells was by Slater et al. in 1986 [24]. Confusion exists regarding the mechanism(s) by which cyclosporin A brings about increased drug effects in resistant (and parent) cells. In some examples, increased effect is apparently associated with increases in intracellular drug accumulation but, in other examples, this does not appear to be the case ([25].

Our data clearly established that these ionophores that once in the membrane are complexed to metal ions are thus either neutral or once positively charged, are able to inhibit the P-gp -mediated efflux of THP-adriamycin. Cyclosporin A which is also a strong metal chelating agent has comparable effect.

**Acknowledgements.** Ths investigation was supported by Université Paris Nord, CNRS and ARC (Association pour la Recherche contre le Cancer).

### References

[1] Bradley, G., Juranka, P.F & Ling, V. (1988) Mechanisms of multidrug resistance. Biochim. Biophys. Acta 948, 87-128.

[2] Gottesman, M.M. & Pastan, I.(1993) Biochemistry of multidrug resistance mediated by the multidrug treansporter. Annu. Rev. Biochem. 62, 385-427.

[3] Dano, K. (1973). Active outward transport of daunomycin in resistant Ehrlich ascites tumor cells. Biochim. Biophys. Acta, 323, 466-483.

[4] Riordan J. R. & Ling, V. (1985). Genetic and biochemical characterization of multidrug resistance. Pharmacol. Ther., 28, 51-75.

[5] Skovsgaard, T. (1978) Mechanisms of resistance to daunorubicin in Ehrlich ascites tumor cells. Cancer Res., 38, 1783-1791.

[6] Juliano, R.L. & Ling, V. (1976). A surface glycoprotein modulating drug permeability in Chinese hamster ovary cell mutants.Biochom. Biophys. Acta, 455, 152-162.

[7] Beck, W.T., Mullen, T.J. & Lanzen, L.R. (1979). Altered surface membrane glycoprotein in Vinca alkaloid-resistant human leukemic lymphoma. Cancer Res., 39, 2070-2076.

[8] Higgins, C.F. (1992) . ABC transporters: from microorganisms to man. Annu. Rev. Biol. 8, 67-113.

[9] Thiebault, F., Tsuruo, T., Hamada, H., Gottesman, M.M., Pastan, I. & Willinham, M.C. (1987) Cellular localization of the multidrug resistance gene product P-glycoprotein in normal tissues. Proc. Natl. Acad. Sci. USA, 84, 7735-7738

[10] Pearce HL, Winter MA and Beck WWT.(1990). Strutural characteristics of compounds that modulate P-glycoprotein-associated multidrug resistance. Advan Enzyme Regulation, 357-372.

[11] Tarasiuk J, Frezard F, Garnier-Suillerot A and Gattegno L. (1989) Anthracycline incorporation in human lymphocytes. Kinetics of uptake and nulear concentration. Biochim Biophys Acta 1013, 109-117.

[12] Frezard F and Garnier-Suillerot A. (1991) Determination of the osmotic active drug concentration in the cytoplasm of anthracycline -resistant and -sensitive K562 cells. Biochim Biophys Acta 1091, 29-35.

[13] Frezard F and Garnier-Suillerot A.(1991) Comparison of the membrane transport of anthracycline derivatives in drug-resistant and drug-sensitive K562 cells. Eur J Biochem 196, 483-491.

[14] Daoud, S.S. & Juliano, R.L.(1989) Modulation of doxorubicin resistance by valinomycin (NSC122023) and liposomal valinomycin in Chinese Hamster Ovary cells. Cancer Res., 49, 2661-2667.

[15] Crifo, C., Capuzzo, E., Cucco, C. Zupi, G. & Salerno, C.(1991) Valinomycin-induced modulation of adriamycin resistance and cationic distributio in MCF-7 cell lines. Biochem. Intern., 25, 593-601.

[16] Sehested, M., Skovsgaard, T. & Roed,H. (1988) The caboxylic ionophore monensin inhibits active efflux and modulates in vitro resistanec in Ehrlich ascits tumor cells. Biochem. Pharmacol., 37, 3305-3310.

[17] Klohs, W.D. & Steinkampf, R.W. (1988). The effect of lysosomotropic agents and secretory inhibitors on anthracycline retention and activity in multiple drug-resistant cells.Mol. Pharmacol., 34, 180-185.

[18] Mariani, MF, Thomas, L, DeFeo B., van Rossum GDV (1989) Effects of monensin on ATP levels and cell functions in rat liver and lung in vitro. J. Membrane Biol. 108, 235-246.

[19] Rotin, D., Wan, P., Grinstein, S., Tannock, I. (1987) Cytotoxicity of compounds that interfere with the regulation of intracellular pH: a potential new class of anticancer drugs. Cancer Res.

47, 1497-1504

[20] Pressman, B.C. (1985) "The discovery of ionophores: an historical account" in Metal ions in biological systems. Sigel, H. Ed., Marcel Dekker, Inc. , New York.

[21] Carver, J.A., Rees, N.H., Turner, D.L., Senior, S.J. & Chowdhry, B.Z.(1992) NMR studies of the $Na^{2+}$, $Mg^{2+}$ and $Ca^{2+}$ complexes of cyclosporin A. J. Chem. Soc., Chem. Commun, 1682-1684.

[22] Haynes, M., Fuller, L., Haynes, D.H. & Miler, J. (1985). Cyclosporin partitions into phospholipid vesicles and disrupts membtrane architecture.Immunol. Lett. 11, 343-349.

[23] Matyus, L., Balazs, M., Aszalos, A. Mulhern, S. & Damjanovich, S. (1986). Cyclosprin A depolarizes cytoplasmic membrane potential and interacts with $Ca^{2+}$ ionophores. Biochim. Biophys. Acta, 886, 353-360.

[24] Slater, L.M., Sweet, P., Stupecky, M. & Gupta, S. (1986). Cyclosporin A reverse vincristine and daunorubicin resistance in acute lymphatic leukemia in vitro. J. Clin. Invest. 77, 1405-1408.

[25] Twentyman (1992). P.R.Cyclosporins as drug resistance modifiers. Biochem. Pharmacol. 43, 109-117.

**Received: September 7, 1993**

